# With axial loading during MRI diurnal T2-value changes in lumbar discs are neglectable: a cross sectional study

**DOI:** 10.1186/s12891-018-1930-0

**Published:** 2018-01-22

**Authors:** L. Torén, H. Hebelka, I. Kasperska, H. Brisby, K. Lagerstrand

**Affiliations:** 1 Department of Radiology, Sahlgrenska University Hospital, Gothenburg, Sweden; 2 Department of Orthopaedics, Sahlgrenska University Hospital, Gothenburg, Sweden; 3 Department of Medical Physics and Techniques, Sahlgrenska University Hospital, Gothenburg, Sweden; 4 Institute of Clinical Sciences, Sahlgrenska Academy, University of Gothenburg, Gothenburg, Sweden

**Keywords:** IVD, Axial loading during MRI (alMRI), T2-value, Diurnal variation, T2 mapping

## Abstract

**Background:**

Axial loading during MRI (alMRI) combined with T2 mapping recently was shown as a promising method to reveal biomechanical intervertebral disc (IVD) characteristics. This feasibility study aims to investigate whether there is a diurnal variation in the IVD T2-value when using alMRI. This is of importance for the planning of when to perform alMRI investigations and for interpretations of alMRI findings in relation to clinical symptoms.

**Methods:**

Six healthy volunteers (30 lumbar discs), were examined with alMRI at three different sessions during 1 day. To be representative for a low back pain cohort in terms of age and IVD degeneration the included participants had a wide age range (27-63y) and all Pfirrmann grades represented. The T2-values were measured in five IVD regions of interest (ROI). The ROIs were equally large in sagittal plane with ROI1 representing anterior parts of the IVD, ROI5 posterior IVD parts and ROI2–4 the parts in between.

**Results:**

T2-values of the entire IVD varied between 38 and 138 ms at 7 am, 33-143 ms at 11.30 am, and 31-147 ms at 4 pm with large regional IVD variations at all time points. No significant alterations of the T2-values over the day were found, neither for the entire IVD (*p* = 0.4) nor for the various ROIs (*p* = 0.2–1.0). Neither when correlated to Pfirrmann grade, any significant diurnal T2-value changes were found.

**Conclusions:**

With alMRI, only minor diurnal T2-value changes were found in the lumbar discs. Nonsignificant and neglectable diurnal changes are advantageous both for research purposes, as well as in the clinical setting, giving comparable and robust data regardless of at what time-point the alMRI is performed.

## Background

In spite of extensive research, trying to establish diagnostic methods for detailed assessment of intervertebral disc (IVD) degeneration and spinal pain predictors, current imaging methods are insufficient. Today, the imaging methods used for low back pain (LBP) have low specificity and lack the capacity to identify which anatomical structures are causing the patients pain [1–3]. Furthermore, conventional clinical imaging do not enable objective IVD degeneration classification neither assess detailed information regarding IVD architecture. Chronic LBP, often associated with IVD degeneration [4–6], is a worldwide main health issue causing large suffering on an individual level and high economic cost for the society [7]. Clinical care would therefore benefit from improved imaging diagnostics regarding degenerative disc disease [2].

Combining quantitative magnetic resonance imaging (MRI) of the lumbar back with axial loading during MRI (alMRI), has been shown to be a promising way to improve the imaging diagnostics of degenerative disc disease, and reveal functional IVD characteristics [8]. The alMRI already play an important role in clinical management, such as within spinal stenosis [9–11] where alMRI can reveal occult nerve root compressions not seen with conventional MRI in the supine position [12]. Furthermore, alMRI has been shown to aggravate pain and to instantaneously induce regional IVD changes in LBP patients [8, 13], with increased T2-values of the anterior annulus fibrosus (AF) and nucleus pulposus (NP) respectively with decreased T2-values of the posterior AF [8]. Quantitative T2-values are known to correlate to hydration grade with an inverse correlation to degeneration grade of the IVD [8, 14]. Quantitative MRI may, thus, enable early detection of global and regional biochemical changes in the IVD that cannot be seen morphologically with qualitative MRI [15].

The IVDs have been demonstrated to have a dynamic behavior over the day. De Puky et al. showed that on a macroscopic level the diurnal load diminished the height of the IVDs with about 1%, which was then restored during rest [16]. They also found that degenerated IVDs did not alter as much in height as non-degenerated IVDs. Qualitative MRI studies of the IVD have proposed that diurnal load alters the hydration grade of various sub-regions of the IVDs [17]. Quantitative MRI of young volunteers has revealed diurnal changes of T2-values in entire IVDs and in different sub-regions of the IVDs [18–21], indicating a dynamic displacement of water between the sub-regions [18, 19]. To our knowledge, it has not yet been investigated if and how the T2-values vary over the day with alMRI.

Since previous studies indicate that the molecular equilibrium in the IVDs might be different in the compressive state and that the compressive force generated by axial load instantly alters the global biochemical IVD composition [8, 22], one may assume that the diurnal behavior of the alMRI T2-values would be different for the compressive state.

This feasibility study aims to investigate whether there is a diurnal variation in the IVD T2-value when using alMRI.

This is of importance for the planning of when to perform alMRI investigations and for interpretations of alMRI findings in relation to clinical symptoms.

## Methods

### Participants

In this cross sectional study six healthy volunteers were examined with alMRI, at three time points during 1 day, with 30 lumbar IVDs analyzed. None of the volunteers suffered from any known medical history of back pain or spine related disease. The inclusion of the volunteers aimed to largely reflect a LBP cohort in terms of age and IVD degeneration (four men, mean age: 38y, age range 27-63y and two women, mean age: 37y, age 28–46y), with all Pfirrmann grades represented.

### Image acquisition

Each volunteer was investigated at three different sessions during the same day. Every session consisted of one MRI scan with the spine in an unloaded position (referred to as uMRI) instantly followed by one scan with alMRI. The first session was at 07 am, the second at 11.30 am and the last at 04 pm. No restrictions regarding activity were imposed before or between sessions. The alMRI was carried out in supine position with a loading device (DynaWell compression device, Dynawell diagnostics AB, Las Vegas, NV, USA), set at half the body weight of the actual volunteer. During uMRI, the lower leg of the volunteer rested on a foam support of 10 cm height, resulting in slight bending of the hip and knee joints. During alMRI, a cushion of approximately 8 cm height was used between the bed and the lumbar back, creating a lumbar lordosis in order to simulate normal upright position [23].

The MR-images were acquired with a Siemens Magnetom Aera 1,5 T scanner (Erlangen, Germany). The scan protocol consisted of a sagittal turbo spin echo (TSE) T1 weighted (w) sequence, a sagittal TSE T2w sequence and an axial TSE T2w sequence. A sagittal multi echo spin echo (SE) T2-sequence was incorporated at the end of the protocol enabling sagittal T2-maps to be calculated. High quality standardized T2-maps (256 × 256 matrix, slice thickness 3.5 mm, FOV 220 × 220 mm^2^, NEX: 1) were finally reconstructed.

### Image analysis

All image analysis were made by a radiology resident, supervised by an experienced radiologist. Degeneration of the IVDs were graded according to the Pfirrmann classification [24]. To enable further image analysis, multiplanar reconstructions were made from the T1w-sequences. 10 mm thick (in order to enable volumetric IVD analysis) midsagittal slices of sagittal TSE T1w-sequences were fused with sagittal SE T2-mapping sequences using software from syngovia (Siemens, Erlangen Germany).

**Fig. 1:**
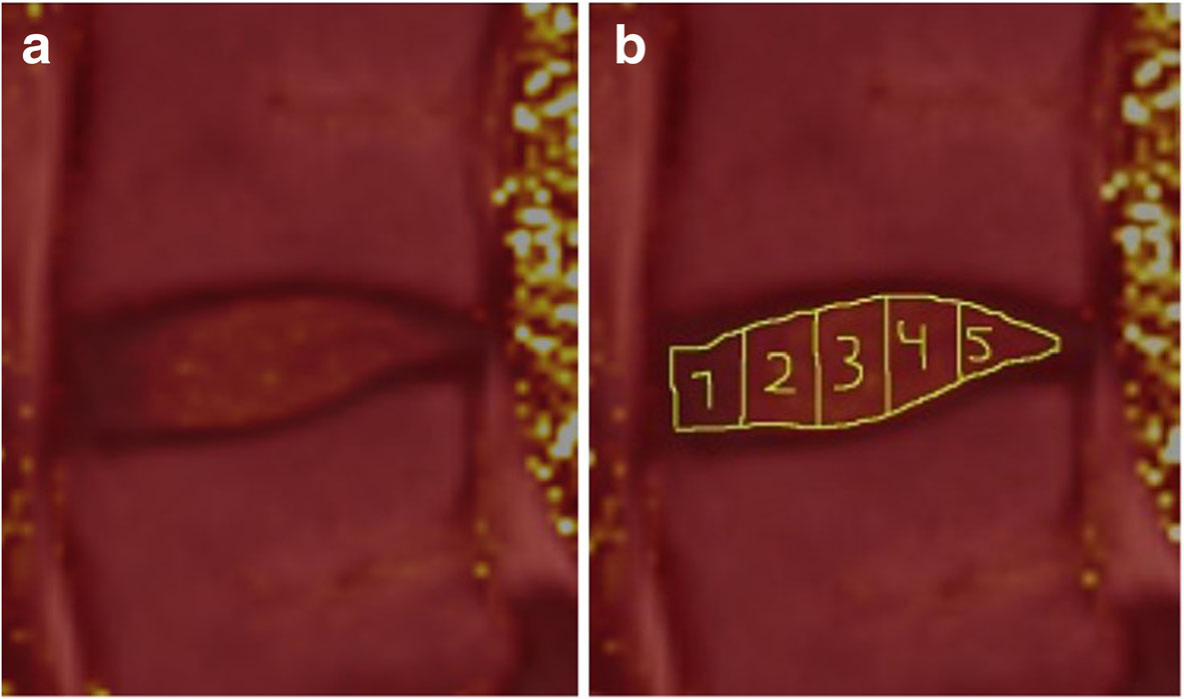
Typical color setting of the images used for the IVD segmentation (**a**). The images, used for IVD segmentation, were derived by fusing T1-weighted images with T2-map images to enhance the borders of the IVDs. The IVDs were divided in to five subregions (ROI1–5), each with different volume but the same sagittal measure (**b**)

The entire IVD was delineated in the sagittal plane using the polygonal region of interest (ROI) tool. Craniocaudally, the IVD was delineated by the endplates and anterioposteriorly by the anterior longitudinal ligament and the posterior longitudinal ligament. The entire IVD was further divided into five volumetric sub-regions with equal length in the sagittal plane, referred to as ROI1 to ROI5. Thus, ROI1 corresponded to the anterior parts of the IVD, ROI5 the posterior IVD parts and ROI2–4 the regions in between (Fig. 1).

The mean T2-time and standard deviation of the mean T2-time were assessed for the entire IVDs and for each sub-region, on both alMRI and uMRI. To ensure reliable measurements, blinded inter-rater agreement was performed before initiating the study. This was done on 5 clinical LBP patients, in which both uMRI and alMRI had been performed. 36 IVDs were measured with approximately half evaluated at alMRI. Inter-rater agreement was substantial for ROI1 and almost perfect regarding ROI2–5 (Table 1) [25, 26]. To test the robustness of the scanning, three repeated scans with alMRI and three repeated scans with uMRI were made on one volunteer at the same session.

**Table 1:** ICC measurements for each ROI

ROI (*n* = 36)	ICC	95% CI
1	0.79	0.62–0.89
2	0.95	0.90–0.97
3	0.99	0.97–0.99
4	0.98	0.96–0.99
5	0.86	0.74–0.99

ICC for inter-rater measurements for each ROI with corresponding 95% CI (confidence interval)

#### Statistics

If not otherwise stated, all T2-values are expressed as mean. The variation was defined as the standard deviation (SD) of the mean. For comparison between differences in T2-values over day, a parametric paired *t* test was applied. Non-parametric Spearman rank correlation were used for analyzing T2-values in groups with different Pfirrmann grades. All tests were two-sided, statistical significance was defined as *p* < 0.05. The software used for analyzing data was IBM SPSS Statistics for Windows, Version 22.0 (Armonk, NY: IBM Corp, USA). Reliability of ROI measurements for inter-rater agreement was performed using Intra-class correlation coefficients (ICC), model 2, with 95% confidence intervals (CI). The coefficients were interpreted using Cronbach’s alpha. Intra-rater agreement has previously been reported [8].

## Results

### alMRI T2-values over the day for the entire IVDs and sub-regions

**Fig. 2:**
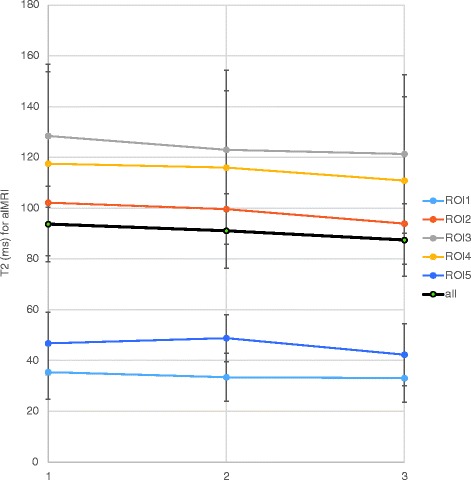
Mean T2-values for the various subregions of the IVD (ROI1–5) with axial loading during MRI (alMRI) at the three separate scanning sessions

The alMRI T2-value slightly decreased over the day in ROI2 to ROI4, from 102 ms (ROI2), 128 ms (ROI3) and 118 ms (ROI4) in the morning to 94 ms, 121 ms and 111 ms, respectively in the afternoon (Fig. 2/Table 2). Thus, small alterations in absolute numbers of alMRI T2-values over the day were discerned. However, no significant changes were found (Table 2), neither for the entire IVDs nor for the separate sub-regions. For all comparative analyzes, the *p*-values were larger than 0.2.

**Table 2:** Mean T2-value at all time instances for all sub-regions

	all		ROI1		ROI2		ROI3		ROI4		ROI5	
	mean	SD	mean	SD	mean	SD	mean	SD	mean	SD	mean	SD
uMRI												
7am	92	15	30	8	88	23	124	28	123	35	59	17
11.30am	88	9	28	9	86	25	115	20	116	23	62	18
4pm	85	12	28	7	82	25	111	24	110	21	61	25
alMRI												
7am	94	15	35	11	102	28	128	28	118	36	47	12
11.30am	91	15	33	9	100	24	123	31	116	30	49	9
4pm	87	14	33	9	94	28	121	31	111	33	42	12
uMRI												
7am vs. 11.30am	*p* = 0.8		*p* = 0.4		*p* = 1.0		*p* = 0.7		*p* = 0.6		*p* = 0.8	
7am vs. 4pm	*p* = 0.5		*p* = 0.4		*p* = 0.7		*p* = 0.4		*p* = 0.2		*p* = 0.6	
11.30am vs. 4pm	*p* = 0.5		*p* = 0.8		*p* = 0.8		*p* = 0.6		*p* = 0.4		*p* = 0.6	
alMRI												
7am vs. 11.30am	*p* = 0.5		*p* = 0.5		*p* = 0.9		*p* = 0.7		*p* = 0.8		*p* = 0.8	
7am vs. 4pm	*p* = 0.4		*p* = 0.9		*p* = 0.6		*p* = 0.5		*p* = 0.6		*p* = 0.2	
11.30am vs. 4pm	*p* = 0.7		*p* = 0.6		*p* = 0.6		*p* = 1		*p* = 0.7		*p* = 0.6	
uMRI - alMRI												
7am vs. 11.30am	*p* = 0.3		*p* = 0.8		*p* = 0.8		*p* = 0.9		*p* = 0.9		*p* = 0.5	
7am vs. 4pm	*p* = 0.4		*p* = 0.8		*p* = 0.8		*p* = 0.5		*p* = 0.2		*p* = 0.5	
11.30am vs. 4pm	*p* = 0.5		*p* = 0.9		*p* = 0.9		*p* = 0.5		*p* = 0.3		*p* = 1	

Mean T2-value (ms) at all time instances for all regions of interest (ROI), both with conventional supine MRI (uMRI) and with axial loading during MRI (alMRI). *P*-value (p) for the T2-value change between all time instances are displayed both with uMRI, alMRI as well as the change between (uMRI-alMRI)

### uMRI T2-values over the day for the entire IVDs and sub-regions

**Fig. 3:**
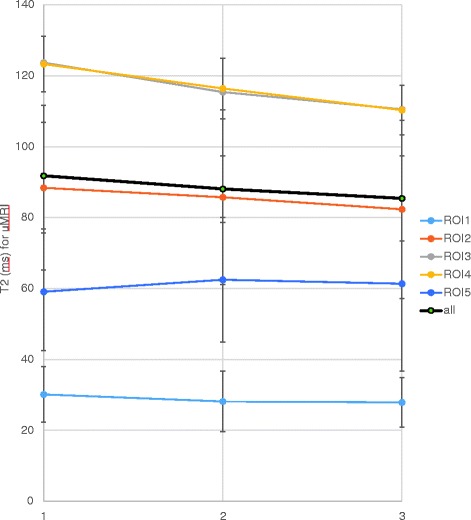
Mean T2-values for the various subregions of the IVD (ROI1–5) sessions with conventional supine MRI (uMRI) at the three separate scanning sessions

Neither for the entire IVDs nor for the separate sub-regions were any significant diurnal alterations found with uMRI. *P*-values larger than 0.2 were found for all comparative analyzes (Fig. 3).

### Comparison of T2-values between alMRI and uMRI

When IVDs were compared between alMRI and uMRI, alterations in T2-values were seen for every sub-region both in the morning, at lunchtime and in the afternoon session. For example, mean T2-value for uMRI in ROI1 was 30 ms (SD 11 ms) compared to 35 ms (SD 12 ms) with alMRI, corresponding to an increase of 17%. Likewise, a decrease of 20% in the mean T2-value was seen in ROI5, where the mean T2-value was 59 ms (SD 19 ms) for uMRI and 47 ms (SD 14 ms) for alMRI.

### alMRI T2-values over the day in relation to Pfirrmann grading

**Fig. 4:**
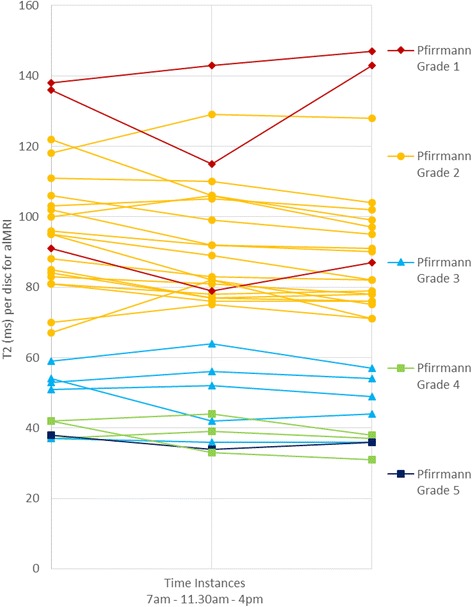
Mean T2-values for the entire IVDs at the three separate scanning sessions

Among the volunteers investigated IVDs with all Pfirrmann grades were represented (Fig. 4), including one IVD with Pfirrmann grade 5. This IVD, localized at L5-S1, was so severely degenerated that only the most 3 ventral ROIs were enabled to be outlined. Most of the IVDs were Pfirrmann grade 2 (Fig. 4). Large diversity of T2-values were detected between all the investigated IVDs. At the morning session, the alMRI T2-values of the entire IVD varied between 38 and 138 ms, at lunch between 33 and 143 ms, whereas the afternoon T2-values varied between 31 to 147 ms. Thus, the difference between the highest and lowest T2-values of the various IVDs were over 300% (Fig. 4). Degenerated IVDs had, in general, lower T2-values. For example, the highest T2-value assessed in an IVD with Pfirrmann grade 5 was 39 ms while the highest value for the entire IVD with Pfirrmann grade 1 was 147 ms. Large regional variations were further seen in non-degenerated IVDs. In a Pfirrmann grade 1 disc for example, the mean alMRI T2-value in the morning was 35 ms in ROI1, 102 ms in ROI2, 128 ms in ROI3, 118 ms in ROI4 and 47 ms in ROI5. In degenerated IVDs, the regional variations were much smaller. In the IVD with Pfirrmann grade 5, the highest T2 value found was 52 ms (ROI3) while the lowest T2 value found was 32 ms (ROI2). When the alMRI T2-values were correlated to Pfirrmann grade, however, no significant changes over the day could be revealed neither on entire IVD level nor within the various sub-regions.

### Reliability measurements

Repetitive scans of the same volunteer showed robustness of the scanning with only minor T2-value variations, however, a 10% alteration was seen at one occasion with alMRI at level L5-S1 between scan 1 (36 ms, SD 14 ms) and scan 2 (40 ms, SD 15 ms). Intra-rater measurements are displayed in Table 1.

## Discussion

The key findings in this study, investigating the diurnal variations of alMRI T2-values, were the minor changes and lack of significant diurnal alterations in the IVDs, neither for the entire IVD nor in its various sub-regions. It was also found that the diurnal alterations in the examined sub-regions of the IVDs were negligible compared to the large differences in mean T2-values between the sub-regions. These findings are of importance both in the clinical setting and for research purposes since alMRI scans can, based on these findings, be performed at any time of the day.

In several studies, the loading of the spine has been shown to affect quantitative MRI parameters of the IVD [8, 22, 27–29]. For example, Nilsson et al. showed that when the IVDs were compressed the T2-values increased in the ventral parts of the IVDs respectively decreased in its posterior parts [8]. Analogously, Stelzender et al. displayed that when the IVDs were unloaded, the T2-values decreased in the ventral part of the IVD and increased in the posterior part of the IVDs [30]. Axial loading compresses the IVDs and introduce another microscopic equilibrium within the IVD, i.e. structural alteration between water, collagen and proteoglycans, different from that of the non-compressed state. Furthermore, Mwale et al. displayed that compressive load of the IVD has a greater impact on quantitative MR parameters than matrix degradation (trypsin digestion) while mechanical IVD properties (compressive modulus, hydraulic permeability and swelling pressure), predominantly are affected by matrix degradation [22]. Thus, alMRI combined with quantitative MRI has the potential to reveal functional IVD characteristics and the current study importantly display that there is no need to standardize when to perform such scans.

Contrary to previous studies on young volunteers, in which diminishing T2-values of NP and increasing T2-values of AF have been displayed [18, 19], no significant diurnal alteration of uMRI T2-values were found. These divergent results could partly be caused by small sample size, but are most likely explained by a higher mean age (38 years) and increased degeneration grade in the current cohort, chosen to be representative for LBP patients. For example, Zhu et al. had a mean age of 25 years in their population (20 subjects) with no subject older than 31 years [18] and Ludescher et al. studied no subject older than 29 years (6 subjects) [19]. The lack of significant diurnal T2-value variations with uMRI found in the current study were expected as age and degeneration grade are established as factors diminishing diurnal variations of T2-values with uMRI [21].

In concordance with previous uMRI studies [18, 21], T2-values of entire IVDs were low in degenerated IVDs and displayed less variation between various sub-regions compared to IVDs without degeneration. These findings are anticipated. Macromolecules, as proteoglycans, are key factors for the water holding capacity of the IVDs. In degenerated IVDs, the amount of proteoglycans relative collagen is reduced in general and in the NP in particular. Hence the capacity of holding water is reduced and also, the water is distributed more equally in the degenerated IVDs which is reflected by lower and more uniform T2-values [18, 21, 31].

The current study delineated the entire IVD, also outermost parts near the endplates. In previous studies, various sub-regions have been delineated as preset rectangular or circular areas [18, 21, 31], not following the proper IVD shape. Furthermore 10 mm sagittal slices with mapped T2-values were analyzed compared to 3–5 mm sagittal slices in previous studies [19, 21, 30]. The increased slice thickness, combined with a delineation covering the entire IVD may reduce errors from local irregularities in the IVDs structure. For example, radial or circumferential fissures in the AF may be filled with gelatinous content from the NP pressed out in the periphery when applying axial load on the IVD, influencing the T2-values in an arbitrary way. The predominant theory is that water is pressed out from NP to the AF during diurnal IVD loading [21]. Theoretically, one could argue that a shift of water from the NP to the periphery may not be as obvious when the entire IVD is delineated, as when using smaller regions, since even the craniocaudal parts of the AF are covered. On the other hand, the delineation in the current study is a more fair way to assess the IVDs. Different ways of delineating the IVDs might however aggravate comparisons between various studies.

This study was designed to enable detection of altered alMRI T2-values during the day. To strengthen the study, three scanning sessions were performed over the day, instead of two scanning sessions per day which have previously been used [18, 19]. Another important strength of this study is that the investigated volunteers are representative of a LBP cohort, covering a large age range with a broad spread with respect to IVD degeneration. As such, the study design makes the results applicable in the clinical setting.

### Limitations

Only a small number of volunteers were included in the present study. Since there is a lack of similar studies, in which quantitative T2-mapping measurements are performed with alMRI, estimation of the sample size was based on expected changes in regional IVD T2-values over the day for unloaded MRI [19, 25]. With a diurnal effect of 10%, a sample size of 5 individuals and 25 IVDs should be sufficient at 80% power with 95% significance and two sided test. One extra healthy volunteer was included in the study to ensure power. With alMRI, however, the effect size was found to be much smaller and, hence, no statistical significance could be verified. Significant or not, the diurnal changes during alMRI were small and thus likely not clinically relevant. Hence, the alMRI method can be considered as robust over the day. Moreover, the included volunteers were well representative for a LBP cohort, with all degenerations grades represented, and thus these data likely are more clinically relevant compared to results from study cohorts with younger mean age and less degenerated IVDs.

### Conclusion

With alMRI, only minor diurnal T2-value changes were found in the lumbar discs. Nonsignificant and neglectable diurnal changes are advantageous both for research purposes, as well as in the clinical setting, giving comparable and robust data regardless of at what time-point the alMRI is performed.

## Abbreviations

AF: Annulus fibrosus
alMRI: Axial loading during MRI
CI: Confidence interval
ICC: Intra-class correlation coefficients
IVD: Intervertebral disc
LBP: Low back pain
NP: Nucleus pulposus
ROI: Region of interest
SD: Standard deviation
SE: Spin echo
TSE: Turbo spin echo
uMRI: MRI with the spine in an unloaded position
w: Weighted
